# Circadian-Related Sleep Disorders and Sleep Medication Use in the New Zealand Blind Population: An Observational Prevalence Survey

**DOI:** 10.1371/journal.pone.0022073

**Published:** 2011-07-18

**Authors:** Guy R. Warman, Matthew D. M. Pawley, Catherine Bolton, James F. Cheeseman, Antonio T. Fernando, Josephine Arendt, Anna Wirz-Justice

**Affiliations:** 1 Department of Anaesthesiology, Faculty of Medical and Health Sciences, University of Auckland, Auckland, New Zealand; 2 School of Biological Sciences, University of Auckland, Auckland, New Zealand; 3 Institute of Information and Mathematical Sciences, Massey University, Auckland, New Zealand; 4 Department of Psychological Medicine, Faculty of Medical and Health Sciences, University of Auckland, Auckland, New Zealand; 5 Centre for Chronobiology, Faculty of Health and Medical Sciences, University of Surrey, Guildford, Surrey, United Kingdom; 6 Centre for Chronobiology, Psychiatric Hospital of the University of Basel, Basel, Switzerland; Vanderbilt University, United States of America

## Abstract

**Study Objectives:**

To determine the prevalence of self-reported circadian-related sleep disorders, sleep medication and melatonin use in the New Zealand blind population.

**Design:**

A telephone survey incorporating 62 questions on sleep habits and medication together with validated questionnaires on sleep quality, chronotype and seasonality.

**Participants:**

Participants were grouped into: (i) 157 with reduced conscious perception of light (RLP); (ii) 156 visually impaired with no reduction in light perception (LP) matched for age, sex and socioeconomic status, and (iii) 156 matched fully-sighted controls (FS).

**Sleep Habits and Disturbances:**

The incidence of sleep disorders, daytime somnolence, insomnia and sleep timing problems was significantly higher in RLP and LP compared to the FS controls (p<0.001). The RLP group had the highest incidence (55%) of sleep timing problems, and 26% showed drifting sleep patterns (vs. 4% FS). Odds ratios for unconventional sleep timing were 2.41 (RLP) and 1.63 (LP) compared to FS controls. For drifting sleep patterns, they were 7.3 (RLP) and 6.0 (LP).

**Medication Use:**

Zopiclone was the most frequently prescribed sleep medication. Melatonin was used by only 4% in the RLP group and 2% in the LP group.

**Conclusions:**

Extrapolations from the current study suggest that 3,000 blind and visually impaired New Zealanders may suffer from circadian-related sleep problems, and that of these, fewer than 15% have been prescribed melatonin. This may represent a therapeutic gap in the treatment of circadian-related sleep disorders in New Zealand, findings that may generalize to other countries.

## Introduction

The primary problem associated with blindness is, of course, the absence of vision. However, a secondary problem which can have a major impact on the health and well-being of blind people is the disruption of their sleep-wake cycles. This problem can result, at least in part, from the inability of light to synchronise (or entrain) their circadian clock to the external day–night cycle.

Of the six circadian-related sleep disorders listed in the ICSD two have particular relevance to the blind: (i) free-running sleep disorder, in which the circadian clock is not entrained to the 24-hour day but ‘free-runs’ with an endogenous period somewhat longer than 24 hours (on average) [1], [2], [3] and (ii) abnormally entrained sleep disorder, in which sleep is entrained to 24 hours but at an incorrect phase angle i.e. sufferers wake either very early (advanced sleep phase disorder, ASPD) or very late (delayed sleep phase disorder, DSPD) with respect to societal norms.

Several case reports [4], [5], [6], [7], [8], [9], [10] and studies in small groups of blind subjects [11], [12], [13] suggest that sleep-wake timing disorders occur in up to 50% of blind patients [13]. A survey of guide-dog owners in the UK proposed a lower incidence of sleep disorders (14%) [14].

The most comprehensive series of published studies are those from the University of Surrey in the United Kingdom [15], [16], [17], [18]. An initial survey of 388 registered blind participants (56 with no conscious perception of light (NLP)) indicated sleep disturbances in 50% of all respondents, and a higher rate (66%) in NLP subjects [19]. This group then went on to demonstrate an increased incidence of abnormal endogenous melatonin rhythms in subjects lacking conscious light perception (NLP) (n = 67) compared to those with conscious light perception (LP) [16].

Despite reports that sleep timing disorders are a problem in the blind, the overall incidence in the blind population compared to the sighted population remains unknown. Central texts such as the International Classification of Sleep Disorders (ICSD) attest to this fact, stating that *“the actual incidence [of free-running sleep disorder] is unknown”*, *“it is thought that over half of totally blind individuals have a circadian rhythm sleep disorder”* and *“≈70% of blind people report sleep disorders.”* [20]

The only large-scale prevalence study of circadian-related sleep disorders in the blind including sighted controls [21] reported a much lower incidence of disorders than the ICSD estimates. In this study, 83% of respondents reported at least one sleep problem but the rate of free-running sleep disorder was low; only 17% of totally blind respondents identified drifting sleep patterns (compared to 8% in the sighted controls). Sleep pathologies which were more prominent in the blind population included shorter sleep duration, increased sleep latency, nocturnal awakenings and daytime somnolence [21].

Case studies and clinical trials in small groups of blind subjects with no conscious light perception (NLP) have shown melatonin to be effective in stabilising sleep time [8], [9], [10] and entraining the biological clock of blind individuals to the 24 hour day [8], [10], [15], [22], [23], [24], [25], [26], [27]. Blind subjects receiving melatonin improved their sleep quality, sleep timing and quality of life [8], [10], [15], [22], [23], [24], [25], [26], [27], [28]. Few safety data exist for the long term use of melatonin, but those case reports that are available suggest no deleterious effects [29].

New Zealand provides a unique opportunity to conduct an epidemiological survey of sleep disorders in the blind. The Royal New Zealand Foundation of the Blind (RNZFB) is the sole organization in New Zealand through which resources are provided to the blind community. The Foundation's database is one of the most comprehensive in the world, with 11,500 members, and listing details of the degree of visual impairment and underlying cause of blindness for all members.

In addition, due to the interest in socioeconomic factors as important determinants of health inequalities, New Zealand has developed a validated “material deprivation” index (NZDep) based on income, employment, qualifications, living space, communication, and car and home ownership [30], which is available for all street addresses in the country. Previous New Zealand studies have shown increasing deprivation on this scale to be associated with health problems, including insomnia [31], [32].

Major health disparities also exist between Māori (the indigenous people of New Zealand) and non-Māori. Māori present an ethnic group in which sleep problems, particularly sleep apnoea and SIDS, are pronounced [31], [32]. Māori suffer from a higher incidence of insomnia compared to non-Māori even after accounting for other factors such as socioeconomic deprivation. The prevalence of circadian-related sleep disorders in Māori remains unknown.

A further advantage of the New Zealand setting is that the use of melatonin is quantifiable. As in Australia and the United Kingdom, melatonin is only available on prescription, which facilitates the accurate determination of overall rates of use, dosage and timing of administration (though buying through the internet and from US sources cannot be controlled).

While free-running rhythms are common in subjects with no conscious light perception (NLP), abnormally entrained rhythms are more common in blind individuals with light perception (LP). However, normally entrained melatonin rhythms have been reported in monocular subjects suggesting that two eyes may not be necessary for photo-entrainment in all situations. Furthermore, illumination of a single eye can result in at least 50% melatonin suppression (melatonin suppression being used as a marker to infer presence of circadian photo-entrainment) and in the absence of one eye there may even be compensation from the remaining eye. Notwithstanding these findings it is important to note that fully sighted people suffer from circadian related sleep disorders in situations of reduced light intensity. This is despite the fact that in the sleep laboratory, candlelight is sufficient to entrain the human circadian clock [33]. Because of our lack of understanding about how much light perception is required for entrainment of the human circadian clock, in the current study we adopt a cautious approach to allocating our study groups. The three groups chosen here were (1) people with reduced light perception (RLP) (no perception of light in one or both eyes); (2) people with light perception in both eyes, and (3) fully sighted people (FS). This approach enables us to identify whether any degree of reduction in light perception results in an increased incidence of sleep disorders.

Here we present the data from a nationwide survey of (i) the prevalence of self-reported sleep disorders in the New Zealand blind community and (ii) the use of sleep medications (including melatonin) to treat these disorders.

## Methods

### Participants

This observational study received ethical approval from the University of Auckland Human Participants Ethics Committee (REF 2006/008).

Based on previous estimates we calculated that 100 participants would be required in each of the three study groups in order to show a statistical difference in the prevalence of sleep disorders between the groups (p<0.05, power 80%). We recruited and surveyed 157 members of the Royal New Zealand Foundation of the Blind (RNZFB) who had no conscious light perception in one or both eyes (defined as our reduced light perception (RLP) group), randomly selected from the membership lists. One hundred and fifty seven members with conscious light perception in both eyes (LP) matched for age, sex and domicile deprivation coding (NZDep) to the RLP group were also surveyed, along with 157 fully-sighted matched controls (FS) selected from the electoral rolls. Overall, two participants withdrew from the study (one in the LP group and one in the FS group) resulting in final respondent numbers of RLP 157, LP 156 and FS 156. Admission to the Foundation requires a full ophthalmic examination confirming a visual impairment of 6/24 vision or a field of vision of 20° in the best corrected eye. Details of these examinations are held on the RNZFB membership records.

The New Zealand domicile deprivation rating (NZDep) is a nationwide scale in which all street addresses are rated on a scale of 1 (most privileged) to 10 (least privileged). Domicile deprivation codes were recorded for all participants in the RLP group, and respondents in the LP and FS groups were matched (to the extent this was practicable) to the RLP group.

Participants in the RLP and LP groups were further broken down into sub-groups based on the level of light perception in each eye. The primary rationale for this was to enable the direct comparison of the prevalence of sleep disorders between those subjects with no light perception (NLP) in either eye, a group previously shown to exhibit sleep problems, and our ‘reduced light perception’ group. There were three sub-groups in the RLP group: NLP-NLP subjects with no light perception in either eye, NLP-LP subjects with no perception of light in one eye and light perception in the other, and NLP-S subjects with no light perception in one eye and sight in the other. Similarly, the LP group had three sub-groups: LP-LP, LP-S and S-S (the last group being those subjects who meet the criteria for admission to the RNZFB but who have some sight in both eyes).

Half of the entire membership of RNZFB (approximately 6,000 people) were sent a letter by the Foundation, informing them of the study and asking them to identify themselves if they did not wish to be contacted. A second letter providing specific information about the study was then sent to 3,000 randomly selected potential participants. In order to obtain a sample size of at least 150 in each group, 781 randomly selected members from the 3,000 potential participants (456 RLP and 325 LP) were subsequently telephoned and invited to participate in the survey. This procedure was repeated for the FS control group. A toll free number was active throughout the study to enable participants to contact the investigators at no cost. In accordance with ethical guidelines informed consent was obtained over the telephone from all participants (as many visually impaired subjects were unable to read) and consents were stored in coded computer voice files.

### Survey

A 30 minute telephone-based survey with 62 questions was developed and piloted on 30 subjects (excluded from the main survey). The refined survey was conducted between February 2006 and May 2007. This survey (see Supporting Information S1) included:

- questions regarding self-reported sleep habits and sleep problems: sleep-apnoea, restless sleep, unconventional sleep times, sleep latency, nocturnal restlessness, early morning wakening, sleepwalking/talking, non-restorative sleep, daytime somnolence, bruxism; and sleep medication use;
- questions designed to identify circadian-related sleep disorders:
  “Do you always experience difficulties sleeping or do your sleep problems occur intermittently with alternating periods of good and bad sleep?”
  “Do these periods of bad sleep occur at regular intervals?”
  “How long, in days, is it between the start of one period of bad sleep and the start of the next period of bad sleep?”
  “How long, in days, does a single period of bad sleep last?”
  “How long, in days, is the period of good sleep in between?”
  “Do you feel like your sleep pattern drifts with respect to the 24 hour day? If yes, can you describe how it drifts?”
  “Do you ever take naps during the day? Or if you are employed or have other commitments, do you have a strong desire to nap during the day?”
  “Do you nap more during periods of bad sleep?”
- the Pittsburgh Sleep Quality Index (PSQI), a validated index which produces a number from 0–21 with higher scores indicating increasing sleep problems (a score of greater than five or more being indicative of poor sleep [34]);
- the Munich Chronotype Questionnaire (MCTQ) [35];
- the Horne-Östberg Morningness–Eveningness Questionnaire (MEQ) [36];
- the extended Seasonal Pattern Assessment Questionnaire (SPAQ+) to screen for Seasonal Affective Disorder (SAD.) [37], [38]

Details of the cause of participant's vision loss, along with their degree of visual impairment, were obtained from the RNZFB membership list and were confirmed by participants at the time of survey. Demographics and lifestyle variables were recorded (including age (years), sex, BMI, ethnicity (see Table 1 for categories), domicile deprivation rating (NZDep), self-reported depression, caffeinated beverages (drinks/day), alcohol consumption (standard units/week), smoking (cigarettes/day) and recreational drug use (Y/N)). Data were entered directly into a purpose designed Microsoft Access database and exported into Microsoft Excel for analysis.

**Table 1:** Demographics of the participants in the survey (total n = 469).

		Reduced Light Perception		Light Perception		Sighted	
		No.	*%*	No.	*%*	No.	*%*
**Total**		**157**		**156**		**156**	
**BMI**	Mean (SE)	26 (0.56)		26 (0.48)		26 (0.40)	
**NZDep**	Mean (SE)	6.21 (0.21)		6.40 (0.20)		4.9 (0.24)	
**Sex**	Male	87	*55*	85	*54*	85	*54*
	Female	70	*45*	71	*46*	71	*46*
**Age**	0–20	17	*11*	17	*11*	17	*11*
	21–40	18	*11*	17	*11*	20	*13*
	41–60	57	*36*	52	*33*	54	*35*
	61–80	42	*27*	44	*28*	43	*28*
	81+	23	*15*	26	*17*	22	*14*
	Mean (SE)	55 (1.84)		56 (1.84)		55 (1.84)	
**Ethnicity**	NZ European	120	*76*	133	*85*	146	*94*
	Maori	24	*15*	12	*8*	6	*4*
	Pacific	4	*3*	4	*3*	0	*0*
	Indian	4	*3*	2	*1*	2	*1*
	Asian	1	*1*	3	*2*	2	*1*
	Other	4	*3*	2	*1*	0	*0*
**Work**	Yes	71	*45*	64	*41*	100	*64*
	No	86	*55*	92	*59*	56	*36*

Data are shown as raw numbers and percentages (where appropriate). Where means are given, the standard error is shown in parentheses.

### Analysis

#### Sleep habits and problems

Subject groups (RLP, LP, FS) were compared with regard to (i) total sleep duration (hours), (ii) sleep timing (using the midpoint of sleep), (iii) chronotype (MCTQ score and MEQ scores) and (iv) PSQI. Covariates in the initial model included: work, ethnicity, NZDep, self-reported depression, and alcohol consumption. A parsimonious model was found using backward elimination (based on the R^2^ criterion). Generalised Additive Models (GAMs) were used to examine possible non-linearities in the explanatory variables, but effects were quantified using General Linear Model (GLM) (using an identity link structure and Gaussian errors).

The number of self-reported sleep complaints reported by individuals within each group were analysed by GLM (log link, quasi-Poisson error). Age (years), sex and BMI were blocked across the groups. The model also controlled for a variety of covariates including ethnicity, deprivation (NZDep), depression, work (days/week) and alcohol consumption (drinks/week).

#### Prevalence data

The RLP, LP and FS groups were initially tested, using χ^2^ tests for homogeneity, for the prevalence of the following factors:

- specific sleep disorders,
- SAD,
- consulting doctor about sleep problems,
- sleep medication use.

If a significant difference between groups was found, it was further investigated using t-tests for differences in proportions (with Bonferroni corrections for multiple comparisons).

#### Software

The calculations were undertaken with SPSS version 12.0.1 (SPSS Inc., Chicago, IL, USA) and the statistical package R [39].

## Results

### Participation and Demographics

Of the total 11,500 members of the RNZFB, we attempted to contact 456 people in the RLP group and 325 people in the LP group. Of these, 197 people in the RLP group and 177 in the LP group were contactable, and mentally and physically able to participate in the survey. Participation rates were high, with 87% and 88%, respectively, completing the survey.

The demographics of the three groups (257 male, 212 female) are shown in Table 1. The mean age of 55 years reflects the demographic of the membership of the RNZFB, with a large number of its members suffering from age-related visual problems such as macular degeneration. The major ethnic group participating in the survey was NZ European. The second largest ethnic group was Māori, with 15% and 8% of respondents in the RLP and LP groups, respectively, identifying their first ethnicity as Māori.

Unsurprisingly, employment rates in the fully sighted control group were higher than either of the blind or visually impaired groups. This difference in employment status was particularly pronounced in the 18–65 year old age range, with 52% and 54% of people in the RLP and LP group working at least one day a week compared to 92% in the FS controls.

While participants in the RLP and LP groups were well matched for domicile deprivation (no evidence of difference in means), the average deprivation rating of FS controls (4.9±0.24, SEM) was lower than the RLP (6.2±0.2, SEM) and LP (6.4±0.2, SEM) groups, indicating a higher standard of living.

The ten most common causes of blindness/visual impairment are shown in Table 2. Traumatic/surgical injury is the most common cause of blindness in the RLP group, accounting for a quarter of the cases, while macular degeneration is the most common cause in the LP group, again accounting for a quarter of the cases.

**Table 2:** Most common causes of blindness/visual impairment.

RLP			LP		
Cause	No.	%	Cause	No.	%
Traumatic/surgical injury	37	24	Macular degeneration	39	25
Glaucoma	15	10	Retinitis pigmentosa	18	12
Congenital	14	9	Congenital	14	9
Retinal detatchment	12	8	Diabetic retinopathy	8	5
Diabetic retinopathy	10	6	Glaucoma	8	5
Macular degeneration	7	4	Stroke/aneurism/blood clot	5	3
Haemorrhage	5	3	Cataracts	5	3
Cataracts	5	3	Traumatic/surgical injury	4	3
Oxygen at birth	4	3	Oxygen at birth	4	3
Retinoblastoma	4	3	Albinism	4	3
Other	44	28	Other	47	30
**Total**	**157**		**Total**	**156**	

Table indicating the ten most common causes of blindness/visual impairment in the two main groups surveyed (RLP – reduced conscious light perception and LP – conscious light perception in both eyes).

No overall differences in psychological complaints or disorders were detected between the three groups. The most frequent psychological complaint in all three groups was depression, with 4% of RLP, 8% of LP and 2% of sighted controls reporting depression.

### Sleep and the Prevalence of Sleep Disorders

#### Sleep habits

**Figure 1:**
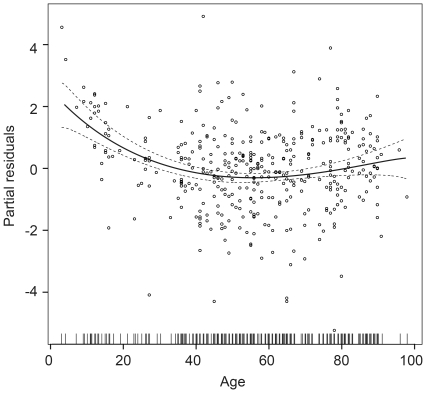
Partial residual plot showing the effect of age on total nightly sleep duration of all subjects (n = 469). No differences in sleep duration were found between the three different subject groups. Dashed lines indicate 95% confidence intervals.

Sleep duration did not differ between the three groups. The mean sleep duration was 7.9±0.2 (SEM) hours in RLP, 8.2±0.18 hours in LP and 7.9±0.01 hours in the FS controls. A non-linear effect of age on sleep duration (irrespective of group) was evident in the data, with a minimum sleep duration at the age of 50 (Figure 1). There was no evidence that self-reported depression had an effect on sleep duration (p = 0.36) or on sleep timing (p = 0.21) as calculated from the MCTQ [35]. A significant difference was, however, found in sleep timing between the RLP and FS groups (p = 0.003); but the magnitude of this difference was small (average mid-sleep: RLP – 2:30 a.m. ±5 min; LP – 2:40 a.m. ±5 min; FS - 2:50 a.m. ±5 min). Mean chronotypes were indistinguishable across the groups with Horne-Östberg scores of 64±0.8, 64±0.7 and 63±0.8 in RLP, LP and FS, respectively (p = 0.413). Participants self-reporting depression had a Horne-Östberg score 5.3 (±1.98) points lower than those not reporting depression but this effect was independent of study group. Daytime napping was reported in 67% of the RLP group compared to 59% of LP and 47% of FS participants. Furthermore, 40% of RLP respondents napped more during periods of bad sleep compared to 21% of LP and 14% of FS participants.

#### Sleep disturbances

The two different measures of sleep disturbance used in the current survey (self-reported sleep disturbance and PSQI) both indicated a higher prevalence of disordered sleep in RLP and LP subjects compared to FS controls.

**Figure 2:**
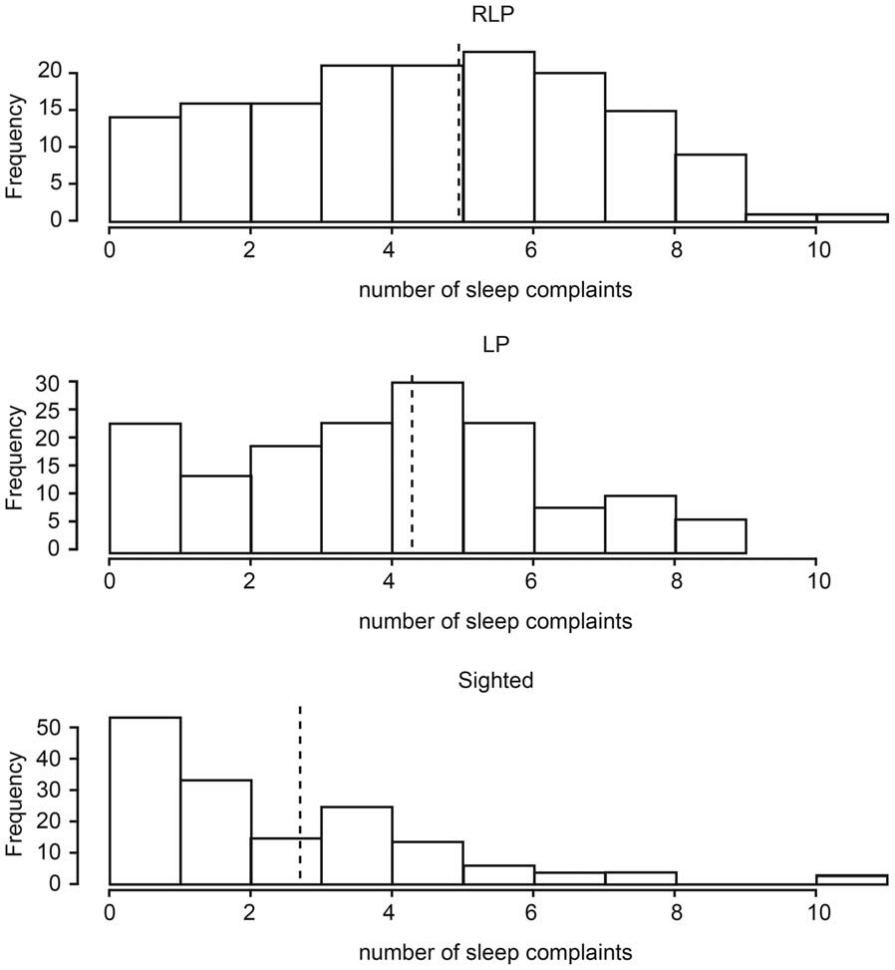
Distribution and frequencies of self-reported sleep complaints across the three subject groups. RLP – reduced light perception, LP – light perception in both eyes, FS – fully sighted. In each case, the mean number of sleep complaints is indicated by a vertical dashed line.

Subjects in the RLP group self-reported experiencing an average of 5.0 sleep disturbances per person compared with 4.3 in the LP group and 2.7 in the FS controls. More sleep disturbances were reported in the RLP (p<0.001) and LP (p<0.001) subjects compared to FS controls. There was also a trend towards more sleep disturbances in the RLP subjects than in the LP group (p = 0.074). The distribution of sleep disturbances in the sighted controls was highly positively skewed. While 91% and 85% of RLP and LP subjects reported having two or more sleep disorders during their life, only 65% of FS controls did so (Figure 2).

There was strong evidence that depression was an important variable when predicting the number of sleep complaints (p = 0.003), with participants with depression reporting on average 32% (95% confidence interval 10% to 58%) more sleep complaints than those without depression. However, this effect did not influence the difference in sleep disturbances between the three study groups.

**Figure 3:**
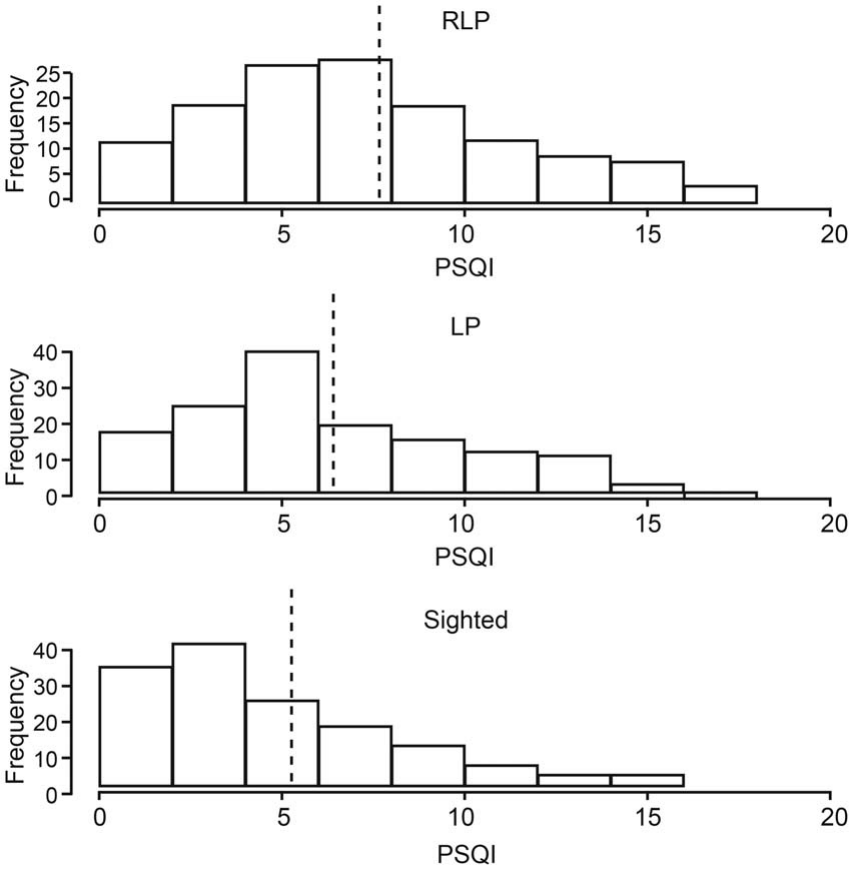
Distribution and frequencies of PSQI scores across the three subject groups. RLP – reduced light perception, LP – light perception in both eyes, FS – fully sighted. In each case, the mean PSQI score is indicated by a vertical dashed line.

PSQI scores confirmed the higher rate of sleep disorders in RLP and LP subjects, with a mean PSQI of 7.6±0.35 in the RLP group compared with 6.7±0.32 in the LP group and 5.3±0.29 in the FS controls (Figure 3). Both the RLP (p<0.001) and LP (p = 0.007) subjects were different from the FS controls, with a trend for PSQI to be higher in the RLP group than in the LP group (p = 0.077). There was no evidence that PSQI was influenced by self-reported depression (p = 0.45). Irrespective of group, each day a person worked per week correlated with a 0.3 point improvement in their PSQI score, however there is no evidence that working had an effect on the prevalence of drifting sleep (p = 0.31).

A breakdown of the incidence of self-reported sleep disorders and PSQI score as a function of total level of vision is given by Table 3.

**Table 3:** Incidence of self-reported sleep disturbances and mean Pittsburgh Sleep Quality Index (PSQI) in the six different sub-groups of the blind and visually impaired respondents.

		NPL-NPL		NPL -LP		NPL-S		LP-LP		LP-S		S-S	
		No.	*%*	No.	*%*	No.	*%*	No.	*%*	No.	*%*	No.	*%*
Total respondents		63		27		67		19		22		115	
**Sleep Problem**													
	Snoring	40	*64*	14	*52*	43	*64*	4	*21*	10	*46*	55	*48*
	Apnoea	9	*14*	8	*30*	8	*12*	2	*11*	1	*4.5*	11	*9.6*
	Restless sleep	40	*64*	18	*67*	40	*60*	11	*58*	14	*64*	65	*57*
	Unconventional sleep times	33	*53*	16	*59*	37	*55*	3	*16*	6	*27*	60	*52*
	Difficulty falling asleep	34	*54*	18	*67*	29	*43*	9	*47*	14	*64*	47	*41*
	Awakenings during sleep	54	*86*	26	*96*	51	*76*	15	*79*	19	*86*	88	*77*
	Inappropriate early morning wakening	40	*64*	21	*78*	33	*49*	13	*68*	9	*41*	51	*44*
	Sleep walk/talk	15	*24*	5	*19*	10	*15*	8	*42*	4	*18*	17	*15*
	Non-restorative sleep	31	*49*	16	*59*	31	*46*	8	*42*	11	*50*	53	*46*
	Daytime somnolence	33	*52*	14	*52*	33	*49*	4	*21*	5	*23*	44	*38*
	Anxiety preventing falling asleep	21	*33*	16	*59*	19	*28*	4	*21*	10	*46*	31	*27*
	Bruxism	5	*8*	6	*22*	8	*12*	4	*21*	1	*4.5*	25	*22*
	Other	15	*24*	7	*26*	19	*28*	1	*5.3*	5	*23*	18	*16*
**PSQI**													
	Respondents	51		24		62		15		20		111	
	Mean PSQI (SE)	8.31	(0.57)	8	(0.81)	6.95	(0.44)	8.27	(0.80)	7.7	(1.0)	6.25	(0.58)

RLP (reduced conscious light perception) is shown in the first three columns; LP (conscious light perception in both eyes), is shown in the second three columns. Data are shown as raw numbers and percentages (where appropriate). Where means are given, the standard error is shown in parentheses.

The different rates of self-reported sleep disorders in RLP and LP subjects compared to FS controls (with higher rates in the RLP and LP groups) were caused, almost exclusively, by problems with sleep timing, insomnia and increased sleep latency (Table 4). In contrast, levels of sleep-disordered breathing, bruxism and sleepwalking/talking were similar across the three groups (Table 4).

**Table 4:** Statistically significant differences in the rates of self-reported sleep disturbances across the three subject groups.

Disorder	RLP-LP	RLP-S	LP-S
**Apnoea**	ns	ns	ns
**Bruxism**	ns	ns	ns
**Sleep walking/talking**	ns	ns	ns
**Restless sleep**	ns	***p<0.001	*** p<0.001
**Difficulty falling asleep**	ns	***p<0.001	*p = 0.033
**Awakenings during sleep**	ns	***p<0.001	p = 0.099
**Anxiety preventing falling asleep**	ns	***p<0.001	* p = 0.03
**Non-restorative sleep**	ns	***p<0.001	* p = 0.003
**Unconventional sleep times**	ns	**p = 0.001	ns
**Inappropriate early morning wakening**	ns	***p<0.001	***p<0.001
**Daytime somnolence**	p = 0.066	***p<0.001	p = 0.066

(RLP – reduced light perception, LP – light perception in both eyes, FS – fully sighted). Asterisks denote significant difference from fully sighted controls (Chi^2^) (* p<0.05, ** p<0.005 ***p<0.001).

When investigating the prevalence of potential circadian-related problems in more depth, it was particularly noteworthy that self-reported drifting sleep (used as an indicator of free-running sleep patterns) was reported in 26% of RLP subjects, 21% of LP subjects and only 4% of sighted controls. Totally blind subjects i.e. those with no light perception in either eye (NLP-NLP), who are the most likely to have free-running sleep disorders, self-reported similar levels of drifting sleep to the overall RLP group (most of whom have light perception in one eye) at 27%.

The number of permanent sleep problems was higher in the FS controls than the two blind/visually impaired groups (46% vs c. 33%) (Table 5). By contrast, intermittent sleep problems were far more common in the RLP and LP groups (Table 5). Regular patterns in intermittent sleep disturbances were most commonly reported in the RLP group. In this group, the mean period between the start of one stretch of bad sleep and the next was 21 days (which, assuming a free-running clock, would equate to an average free-running period of 25.14 hours).

**Table 5:** Sleep disturbances in respondents with one or more sleep problem.

	RLP	LP	S
**Permanent sleep problems**	33%*	32%*	46%
**Intermittent sleep problems**	67%**	68%**	54%
**Desire to sleep at unconventional times**	55%**	43%**	32%
**Regular patterns in sleep disturbances**	12%**	7%**	2.6%
**Drifting sleep patterns**	26%**	21%**	4%
	(27% NLP-NLP)		
**Mean period of sleep disturbances**	21 days	12 days	14 days

Details of the nature and timing of occurrence of sleep disturbances in respondents reporting they had experienced one or more sleep problems (n = 155 RLP, n = 147 LP, n = 134 FS). Asterisks denote significant difference from fully sighted controls (Chi^2^) (* p<0.05, **p<0.005).

The RLP and LP groups were 2.41 (odds ratio 2.41, 95% confidence interval 1.5 to 3.9) and 1.63 (odds ratio 1.63, 95% confidence interval 1.0 to 2.61) times more likely to have unconventional sleep timing problems, respectively, than the FS control group. Drifting sleep was 7.3 (odds ratio 7.3, 95% confidence interval 3.2 to 19.9) and 6.0 (odds ratio 6.0, 95% confidence interval 2.6 to 16.4) times more likely to occur in the RLP and LP groups (respectively) than in the FS controls.

#### Seasonal Affective Disorder

Rates of Seasonal Affective Disorder (SAD) and sub-syndromal Seasonal Affective Disorder (SSAD) (as determined by SPAQ+) in the sighted group were similar to those obtained from previous studies [38], [40]. While total rates of SSAD plus SAD were similar in the three groups (RLP 12.7%, LP 14.7% and S 10.2%), the data revealed a trend towards an increased rate of the more serious SAD diagnosis in RLP subjects (5%) compared to the LP (1.9%) and FS (0%) groups. A latitudinal cline in SAD rates was not apparent within the range that is found in New Zealand (35.08°S to 46.6°S).

#### Sleep Disturbances in Māori

Five percent of the members of the RNZFB identify their ethnicity as Māori. Rates of Māori participation in the survey were high at 11%. In the RLP and LP groups, rates of Māori participation were 15% and 8%, respectively. On average, the PSQI score of Māori was 1.96 points higher than non-Māori. Fifty-eight percent of all Māori RLP respondents (14 people) and 25% of Māori LP respondents (3 people) indicated drifting sleep. The odds ratio for drifting sleep in RLP and LP Māori compared to NLP and LP non-Māori is thus 3.7 (95% confidence interval 1.7 to 7.5).

#### Prevalence of Sleep Medication Use

In order to assess the severity of sleep problems, participants were asked whether they had consulted a doctor about them. Once again, rates were much higher in the RLP and LP groups (39% and 31% of people reporting sleep problems had consulted their doctor about treatment) compared to 17% of FS controls (there was strong evidence of differences between RLP and FS, and LP and FS groups, p<0.005).

Of the people seeking treatment from their doctor for their sleep disturbances, the vast majority (90% RLP, 75% LP and 65% FS) were prescribed medications to help them sleep. The most commonly prescribed sleep medication was Zopiclone, followed by low dose benzodiazepines and tricyclics (Table 6). Melatonin was prescribed infrequently: 4% of RLP, 2% of LP and 0% of FS subjects were prescribed melatonin. This corresponds to a maximum of 11% (n = 6) (in the RLP group) of the prescribed sleep medication (Table 6). Of those people who reported drifting sleep (i.e. potential free-runners), a maximum of 15% (again in the RLP group) had been prescribed melatonin.

**Table 6:** Sleep medications prescribed to people presenting to their General Practitioner.

Treatment	RLP	LP	FS
Zopiclone	52%	46%	42%
Benzodiazepines	8%	12%	4%
Tricyclics	3%	10%	15%
Melatonin	11%	6%	0%
Melatonin use as a percentage of the people reporting drifting sleep	15%	9%	0%
Overall Melatonin Use	4%	2%	0%

Medications received by people presenting to their doctor asking for treatment for a sleep disorder. Total medication rates were n = 55 (RLP), n = 36 (LP) and n = 17 (FS). Prescription of the three most common sleep medications (in white) are shown as a percentage of the total number of people receiving treatment for their sleep disorder. Melatonin use is shown (last two rows) as a percentage of the number of people in each group reporting drifting sleep and as a percentage of the entire sample.

## Discussion

The paucity of data on the prevalence of sleep disorders in the blind, and specifically circadian-related sleep disorders, makes it difficult to put into context the extent of these problems, and the importance of research designed to provide effective treatment. Those blind individuals with circadian-related sleep disorders who are fortunate enough to receive appropriate chronotherapeutic treatment from a clinician with a sound knowledge of circadian biology (or by virtue of participating in a research trial), report vast improvements not only in objectively and subjectively measured sleep but also in their quality of life [8], [10], [16], [23], [25], [26], [28].

Here, our aim was to extend current international efforts to understand the prevalence of sleep disorders in the blind in New Zealand, a first-world country with a small well-educated population of four million people. We believe these data give useful insight into the extent of circadian-related sleep problems in the blind in New Zealand, and may also provide adequate statistics for use in larger Western countries (such as Britain and the US) where the organizational structures through which resources are delivered to blind and visually impaired people are not as homogeneous.

Despite a lower sample size than some previous studies (14, 21) our study is novel in that participants in all three groups were matched for domicile deprivation rating, something not possible in previous surveys. In addition, we included specific questions on chronotype and on sleep medication and melatonin use.

The strong non-linear influence of age on sleep duration (irrespective of level of vision) is in line with previous findings that sleep duration shortens with age. No significant differences in sleep habits were noted between the fully sighted and blind/visually impaired group and, unlike the survey by Leger [21], no truncation of total sleep duration in the blind/visually impaired group was seen. The data did, however, indicate a far higher incidence of sleep disorders in the blind and visually impaired population, with the RLP group averaging twice as many sleep problems as the FS controls (p<0.001), along with poorer sleep. While the number of sleep complaints in depressed patients was elevated across all groups, self-reported depression did not account for the differences between groups and did not influence the overall rates of sleep complaints. Mean global PSQI scores of FS subjects were at the upper limit of ‘good sleepers’ at 5.3 (defined as a score of <5 [34]), while RLP at 7.6 and LP at 6.7 were sleeping worse than the sighted controls. The source of these sleep problems is not caused by differences in rates of depression, respiratory-related sleep problems, grinding teeth or sleepwalking/talking, but can be attributed almost entirely to problems of sleep timing and sleep onset insomnia (see Table 4).

Identifying sleep phase disorders (such as ASPD and DSPD) by survey (Horne-Östberg or MCTQ) is commonplace. It is deemed far more difficult to identify free-running sleep disorder without objective assessments of sleep timing and a marker of the circadian clock such as the melatonin rhythm [16], which is obviously not possible in the context of a survey. Our efforts to conduct a telephone-based survey were justified in the main by being able to describe exactly to participants what we meant by drifting sleep patterns. Specific questions on drifting sleep, the intermittent or permanent nature of sleep disturbances, and the period of time between episodes of poor sleep were added with the aim of ensuring that we could robustly determine the presence or absence of a free-running sleep disorder. Through this technique, we believe we have obtained as accurate an assessment of the prevalence of self reported free-running sleep disorder as is possible without objective measures of sleep timing and circadian phase. We consider that the higher incidence of identified ‘free-run’ in our blind and visually impaired population (25% RLP, 21% LP), compared to the 17% found by Leger [21], and the lower rates in the sighted population (FS 4% compared to 8% by Leger), to be a true reflection of the extent of this disorder. We speculate that the divergent rates are due to strengths in our study design and our focus on questions aimed to identify free-run clearly.

The high levels of intermittent sleep disturbances in the RLP group (Table 5), the fact that these epochs of poor sleep were accompanied by increased daytime napping, and the period of recurrence of poor sleep further support the contention that the sleep-wake cycle is free-running in this group. The mean period (in days) between stretches of poor sleep for the RLP group, at 21 days (which equates to an average free-running period of 25.14 hours), was within the range of previously documented free-running periods for the RLP group [12], [15], [28], [41]. In the LP and FS subjects, the average reported duration was much shorter (12–14 days), which would equate to either a free-running period which is unusually long (25.85 hours), or short (22.15 hours). The only way of confirming a free-run in these people would be to record a marker of their circadian phase over several days or weeks (see, for example, [15]).

Rates of SAD and SSAD in our sighted population were similar to those reported from Australia and Europe [38], [40]. As might be expected for a relationship of seasonality with long winter nights, previous studies have shown that the prevalence of SAD and SSAD is correlated with latitude. However, the latitudinal cline for winter depression stops at about 38°N, which could explain why prevalence studies farther north have failed to find such an effect [42]. Similarly, in the Southern Hemisphere, New Zealand (35.08°S to 46.6°S) only covers some of these lower latitudes, and so no latitudinal cline should be expected (at least, not in this small sample).

The trend towards more serious seasonal problems in the RLP group (evidenced by the higher level of SAD vs SSAD) is interesting in terms of the association of delayed sleep or delayed circadian phase in SAD. In general, individuals with delayed phase are more likely to be depressed or, conversely, depression is often associated with delayed phase [43]. The blind and visually impaired have a double biological vulnerability (not to mention the psychological problems). With reduced light input to the circadian clock comes the tendency to drift to a phase delayed position. In winter, light exposure is even less so they drift even more. Thus, the circadian sleep abnormality coexists with or perhaps promotes SAD symptoms in susceptible individuals. Our data suggest that SAD may be an additional problem in people with impaired light perception. The finding that participants self-reporting depression had a five point lower Horne-Östberg score (more evening chronotype) than those not reporting depression also fits the associations previously found.

Previous surveys of sleep disorders in New Zealand [31], [32] have demonstrated a higher rate of insomnia symptoms among Māori compared to non-Māori. Being Māori is an independent risk factor for reporting sleep complaints including multiple awakenings, early morning awakening and having current sleep problems. Paine and Gander point out the implications for planning services that address health inequalities in New Zealand [32]. The data from our study indicate that attention should be paid to the probability that blind Māori may be a subset of the blind population who suffer disproportionately from circadian-related sleep disorders.

The severity of sleep problems in blind and visually impaired New Zealanders was such that twice the number of people in the RLP and LP groups had presented to the doctor with a sleep complaint compared to FS controls, and, consequently, they had higher rates of sleep medication prescription. In contrast, the rates of prescribing melatonin were extremely low. Zopiclone was the most frequently prescribed sleep medication in all three groups, followed by low dose benzodiazepines and tricyclic antidepressants. Melatonin was used by only 4% of people in the RLP group and 2% of people in the LP group. None of the participants in our survey reported receiving any detailed information about timing their use of melatonin (other than “take it at bedtime”) and two participants were told to stop taking melatonin after a month, even though they found melatonin greatly helped their sleep timing problems, as long term use was deemed dangerous (in analogy with conventional sleep medication).

Safety data on melatonin use are limited. However, reports of adverse effects of short term melatonin use are rare [44]. A Cochrane review on the use of melatonin for jet lag does suggest caution in patients with epilepsy or who are taking warfarin [45]. A recent study of melatonin treatment in children with autism spectrum disorders also indicated an increase in morning sleepiness and nocturnal enuresis [46]. Case studies of long term melatonin use (over years) have not identified any adverse effects [29].

Melatonin's actions as a mild sleep promoting drug which works to shorten sleep latency are known to many. Its primary action as an entraining drug (or chronobiotic) is virtually unknown outside the scientific community. As a consequence of this ignorance, melatonin may be taken at inappropriate times and too-high doses, with the result that it provides little or no help as an entraining agent, and may even lead to the aggravation of the sleep disorder. The extremely low rate of melatonin use in New Zealand almost certainly reflects a lack of knowledge of melatonin's properties by general practitioners, and wariness about safety, appropriate dosage and timing which stem from not knowing the basics of circadian physiology. One of the potential benefits of this study would be programmes to educate New Zealand practitioners on the appropriate and effective use of melatonin to alleviate sleep timing problems in the blind population.

A limitation of our study is that the results obtained are subjective and participants' sleep disorders were not clinically diagnosed. However, the empirical results we have obtained show that the self-reported incidence of sleep disorders, and sleep timing disorders in particular, is far higher in the blind population compared to the sighted population of New Zealand.

While previous studies have indicated a higher incidence of sleep disorders in people with no perception of light (NLP) compared to those with light perception, here we have shown that a reduction in light perception (rather than a total absence) results in increased sleep disruptions. Self-reported sleep disturbances in people with reduced light perception are as high as those with no light perception. Extrapolations from the current study suggest that in excess of 3,000 blind and visually impaired New Zealanders may suffer from circadian-related sleep problems and that less than 15% of them have been prescribed melatonin to combat their sleep timing problems. This represents a therapeutic gap.

The provision of resources by the RNZFB enables many New Zealanders with vision disabilities to avoid isolation, hold down jobs, and participate fully in our community. Efforts to provide resources to improve their quality of life would be greatly enhanced by addressing the sleep timing problems that are evidently common in this group.

## Supporting Information

Supporting Information S1 **Survey questionnaire.**
(MHT)
